# Association Study of Genetic Variants in Calcium Signaling-Related Genes With Cardiovascular Diseases

**DOI:** 10.3389/fcell.2021.642141

**Published:** 2021-11-29

**Authors:** Sen Li, Zhaoqi Jia, Zhang Zhang, Yuxin Li, Meihui Yan, Tingting Yu

**Affiliations:** School of Life Sciences, Beijing University of Chinese Medicine, Beijing, China

**Keywords:** genetic polymorphism, calcium, cardiovascular diseases, cardiovascular risk factors, PheWAS

## Abstract

**Background:** Calcium ions (Ca^2+^) play an essential role in excitation–contraction coupling in the heart. The association between cardiovascular diseases (CVDs) and genetic polymorphisms in key regulators of Ca^2+^ homeostasis is well established but still inadequately understood.

**Methods:** The associations of 11,274 genetic variants located in nine calcium signaling-related genes with 118 diseases of the circulatory system were explored using a large sample from the United Kingdom Biobank (*N* = 308,366). The clinical outcomes in electronic health records were mapped to the phecode system. Survival analyses were employed to study the role of variants in CVDs incidence and mortality. Phenome-wide association studies (PheWAS) were performed to investigate the effect of variants on cardiovascular risk factors.

**Results:** The reported association between rs1801253 in β1-adrenergic receptor (ADRB1) and hypertension was successfully replicated, and we additionally found the blood pressure-lowering G allele of this variant was associated with a delayed onset of hypertension and a decreased level of apolipoprotein A. The association of rs4484922 in calsequestrin 2 (CASQ2) with atrial fibrillation/flutter was identified, and this variant also displayed nominal evidence of association with QRS duration and carotid intima-medial thickness. Moreover, our results indicated suggestive associations of rs79613429 in ryanodine receptor 2 (RYR2) with precordial pain.

**Conclusion:** Multiple novel associations established in our study highlight genetic testing as a useful method for CVDs diagnosis and prevention.

## Introduction

Calcium ions (Ca^2+^) play an essential role in excitation–contraction coupling (EC coupling) in the heart. Dysregulation of intracellular Ca^2+^ level contributes to the abnormal cardiac contractile function during the pathogenesis of cardiovascular diseases (CVDs), such as coronary artery disease and arrhythmias. For instance, Ca^2+^ are involved in the initiation of delayed after-depolarization (DAD) and early after-depolarization (EAD), which mediate the initiation of arrhythmias (Clusin, 2003). The regulation of calcium-handling proteins in a well-coordinated manner is required for Ca^2+^ homeostasis in cardiomyocytes (Eisner et al., 2017). During cardiac action potential, the voltage-gated calcium channels (VGCCs) mediate an influx of external Ca^2+^ which in turn activate ryanodine receptors (RYRs) to release more Ca^2+^ from the sarcoplasmic reticulum (SR). The elevated cytosolic Ca^2+^ cause structural changes in myosin filaments that induce the contraction of muscle fibers. After contraction, Ca^2+^ can either be pumped back to the SR or pumped out of cardiomyocytes through sarco/endoplasmic reticulum Ca^2+^-ATPase (SERCA) or sodium–calcium exchanger (NCX), respectively (Eisner et al., 2017). The activities of these crucial Ca^2+^-handling proteins can be modulated by β-adrenergic signaling and endogenous regulators, such as calmodulin (CALM) and phospholamban (PLN) (Frank and Kranias, 2000; Soltis and Saucerman, 2010). The information of the genes encoding these proteins are summarized in Supplementary Table 1.

Due to the importance of the abovementioned Ca^2+^-handling proteins in maintaining a proper heart function, genetic polymorphisms of calcium signaling-related genes have been proposed to be associated with the pathogenesis of a certain type of CVDs. For example, a genome-wide association studies (GWAS) study has revealed the critical role of Ca^2+^-handling proteins in the myocardial repolarization reflected by the QT interval, linking polymorphisms in Ca^2+^-handling genes to arrhythmias (Arking et al., 2014). However, CVDs represent a group of disorders involving the heart and blood vessels, and the potential associations of genetic polymorphisms in Ca^2+^ homeostasis regulators with different types of CVDs are still inadequately understood. Thus, we performed this hypothesis-driven study to systematically explore the potential associations between 11,274 genetic variants located in 9 calcium signaling-related genes and 118 diseases of the circulatory system using a large sample from the United Kingdom Biobank (UKBB) (*N* = 308,366). For the identified genetic variants in genotype–clinical outcome associations, we also conducted a phenome-wide association study (PheWAS) to examine their potential role in modulating cardiovascular risk factors, including blood pressure and biomarkers.

## Materials and Methods

The United Kingdom biobank recruited participants aged 40–69 years between 2006 and 2010, and the enrolled sampling persons completed a series of questionnaires and measurements at one of the 22 assessment centers in the United Kingdom. Blood samples were also collected at the time of entry. Around 50,000 samples were genotyped on the BiLEVE Axiom chip, while the rest of the genotyping was performed by the United Kingdom Biobank Axiom array, and these two chips had over 95% markers overlapped (Weng et al., 2017). Phasing and imputation were then conducted, and detailed genotyping and imputation procedures could be found elsewhere (Bycroft et al., 2017). We used the imputed variants for analysis and only included those passing Hardy–Weinberg equilibrium (HWE) test, and with minor allele frequency (MAF) ≥ 0.01, information scores (INFO) ≥ 0.7, and genotyping rate ≥ 90%. For 488,263 participants who had genetic data, we removed individuals who were of non-European ancestry, with sex discrepancies, high heterozygosity/missing rate, sex chromosome aneuploidy, or within a third-degree relatedness reflected by kinship coefficient. Participants who withdrew consent or had insufficient information of covariables and inpatient records were also excluded, leading to a final population of 308,366.

PheWAS R package was employed to examine the associations between each of 11,274 genetic variants and 118 outcomes in the disease category of the circulatory system (Denny et al., 2010). Participants’ clinical outcomes were obtained through the national electronic health records with the International Classification of Diseases (ICD) codes. ICD codes are designed primarily for billing and management purposes, and some of the ICD codes represent a single sub-phenotype of a disease, which are not independent phenotypes (Denny et al., 2010; Rahimi et al., 2019). Thus, ICD-9/10 codes from individual-level hospital episode data were mapped to phecodes according to PheWAS Catalog (Denny et al., 2013). Cases were individuals having at least one record for specific phecodes, and controls were defined as participants who were negative for the investigating phecode and related phecodes. Phecodes with cases exceeding 200 were included in the current study to obtain a reasonable power for detecting the genotype–phenotype associations (Verma et al., 2018). The logistic regression was performed to study the potential genotype–clinical outcome association. Age and sex were treated as covariables, and the first five principal components (PCs) were also adjusted for population structure. For multiple testing correction, the Bonferroni method assumed the tests of association performed were independent. However, the variants within a gene might be highly correlated through linkage disequilibrium, and diseases from the same category might also be related to each other, making Bonferroni correction too conservative. Thus, we estimated the numbers of independent variants and phenotypes by PLINK pairwise pruning methods (Tcheandjieu et al., 2020) and PhenoSpD package (Zheng et al., 2018), respectively, which suggested 2,790 independent variants and 96 independent phenotypes. The *p*-value threshold was then calculated as 0.05/(2,790 × 96) = 1.87 × 10^–7^ to define significant association. For the associations surviving a less restrictive threshold (defined as suggestive associations) of 0.05/(the number of independent variants in a gene × 96) (Supplementary Table 1), the GWAS catalog was searched to replicate our results (MacArthur et al., 2017; Tcheandjieu et al., 2020).

For mortality analyses, death information, including the date and cause of death, was obtained from the death registries, and follow-up time was calculated from the date of participants’ first visit to the assessment center to the date of death or the last follow-up date (July 31, 2020). Disease-related mortality was defined when the investigating disease was a primary or contributory cause of death (Batty et al., 2019). For disease incidence analyses, participants with disease onset before the start date were excluded from the cohort, and the person-time for each individual was censored at the date of first disease occurrence, date of death, or the last follow-up date (June 30, 2020 in England, October 31, 2016 in Scotland and February 29, 2016 in Wales), whichever came first. R was used to generate survival curves.

Phenome scan analysis tool (PHESANT) is an opensource tool that can scan the relationships between the variable of interest and a large range of phenotypes and traits in UKBB (Millard et al., 2018; Ripatti et al., 2019). We used PHESANT to scan the causal effect of identified genetic variation on various continuous variables, including blood pressure, birth weight, and blood biochemical indexes. Inverse normal rank transformation was performed for continuous variables before regression to ensure their normality. Age, sex, genotype chip, and the first 10 PCs were used as covariables for adjustments. The conservative Bonferroni corrected *p*-value of 0.05/1,646 = 3.04 × 10^–5^ was employed as the threshold for evaluating the results.

## Results

**FIGURE 1:**
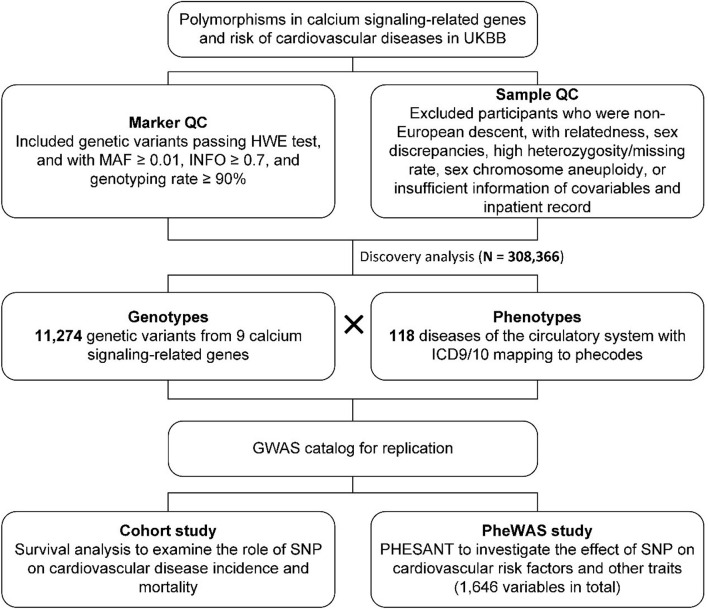
Workflow diagram for discovery and validation of associations between genetic variants in calcium signaling-related genes and CVDs.

**FIGURE 2:**
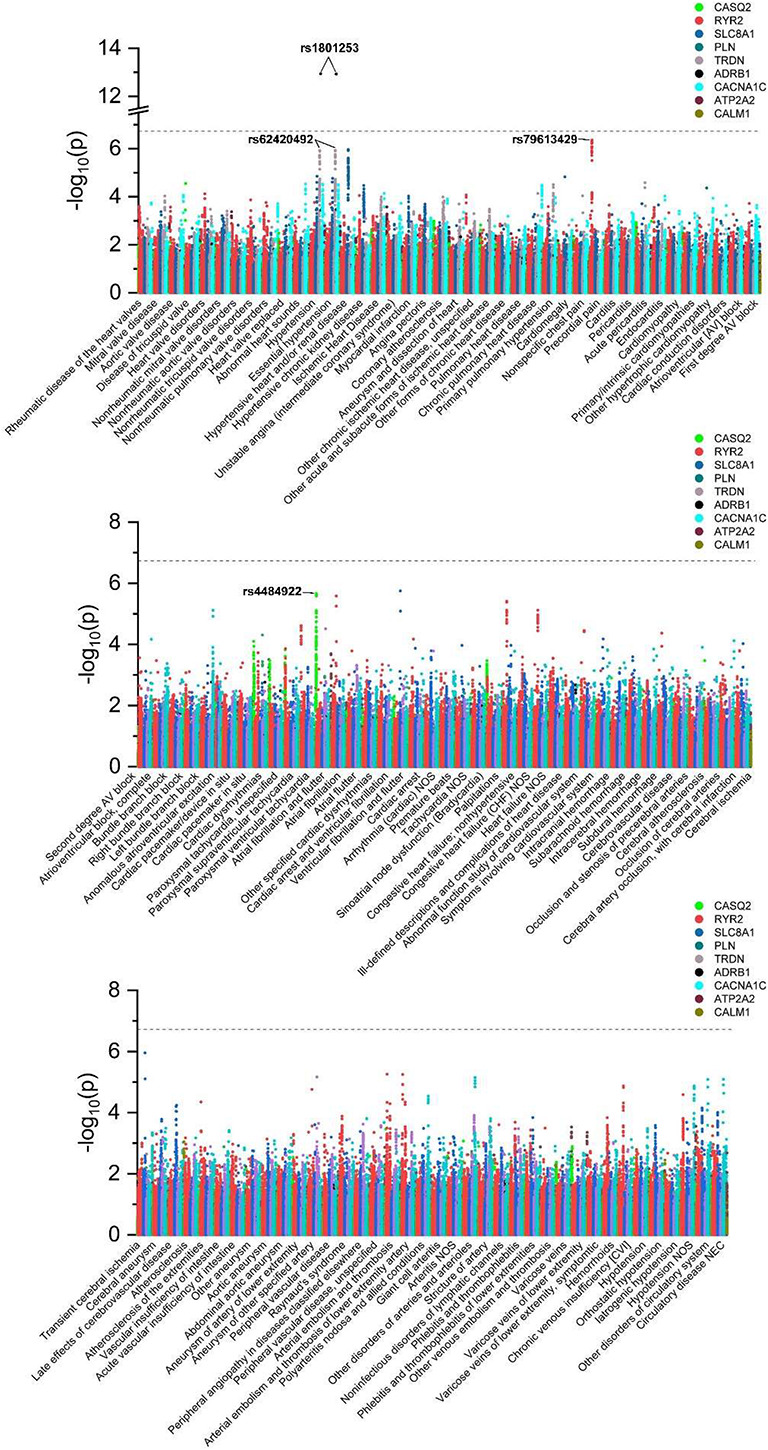
Manhattan plots showing *p*-values of all tested associations between 11,274 genetic variants and 118 diseases of the circulatory system. The threshold indicating a significant level of association is marked by a dotted line. The leading SNPs of significant or suggestive genotype–phenotype associations are annotated.

Demographics of the unrelated UKBB participants involved in the current study are presented in Supplementary Table 2. The number of genetic variants in each of nine calcium signaling-related genes and the description and number of cases for each of 118 diseases of the circulatory system (in terms of phecodes) are provided in Supplementary Tables 1, 3, respectively. To systematically investigate the potential associations between all 11,274 genetic variants and all 118 clinical outcomes, association tests were performed (Figures 1, 2), and the results indicated significant associations between rs1801253 in β1-adrenergic receptor (ADRB1) and hypertension/essential hypertension. Moreover, 11 additional associations reached the suggestive level of significance. Due to the high levels of pairwise linkage disequilibrium (LD) of single nucleotide polymorphisms (SNPs) in one gene (Supplementary Table 4), only the leading SNPs with the smallest *p*-value in genotype–phenotype associations are shown in Table 1. It is worthy of note that the association between rs62420492 in TRDN and hypertension/essential hypertension did not reach the suggestive level after further adjusting the status of overweight (BMI ≥ 25 kg/m^2^), smoking, and drinking as covariables in the model (Supplementary Table 5). We next searched the GWAS catalog to replicate our finding, and the results indicated the association between rs1801253 (or its proxy in high LD with R^2^ > 0.8) and blood pressure had been reported, and the direction of effect was consistent with our observations (Supplementary Table 6). rs4484922 and rs4074536 in calsequestrin 2 (CASQ2) gene were in high LD (Supplementary Table 4) and were reported to be associated with QRS duration and atrial fibrillation, which was also replicated by our finding (Table 1 and Supplementary Table 7). However, the suggestive association of rs79613429 in ryanodine receptor 2 (RYR2) with precordial pain was not found in the GWAS catalog, representing novel genotype–phenotype association.

**TABLE 1:** The associations between SNPs and CVDs that reach significant or suggestive level.

**Gene**	**SNP**	**Minor/reference allele**	**MAF**	**Phenotype**	**N total**	**N cases**	**OR (95% CI)**	***p***
ADRB1	rs1801253	G/C	0.26	Hypertension	303,077	80,845	0.95 (0.94–0.96)	1.15E-13
ADRB1	rs1801253	G/C	0.26	Essential hypertension	302,963	80,731	0.95 (0.94–0.96)	1.17E-13
CASQ2	rs4484922	C/G	0.29	Atrial fibrillation and flutter	290,757	14,954	0.94 (0.91–0.96)	2.17E-06
RYR2	rs79613429	A/C	0.07	Precordial pain	281,944	4,363	1.22 (1.13–1.33)	4.39E-07
TRDN	rs62420492	G/T	0.14	Essential hypertension	306,040	81,518	0.96 (0.94–0.97)	1.16E-06
TRDN	rs62420492	G/T	0.14	Hypertension	306,157	81,635	0.96 (0.94–0.97)	1.20E-06

*SNP, single nucleotide polymorphism; MAF, minor allele frequency; N, number; OR, odds ratio; 95% CI, 95% confidence interval; ADRB1, beta-1 adrenergic receptor; CASQ2, calsequestrin 2; RYR2, ryanodine receptor 2; TRDN, triadin.*

**FIGURE 3:**
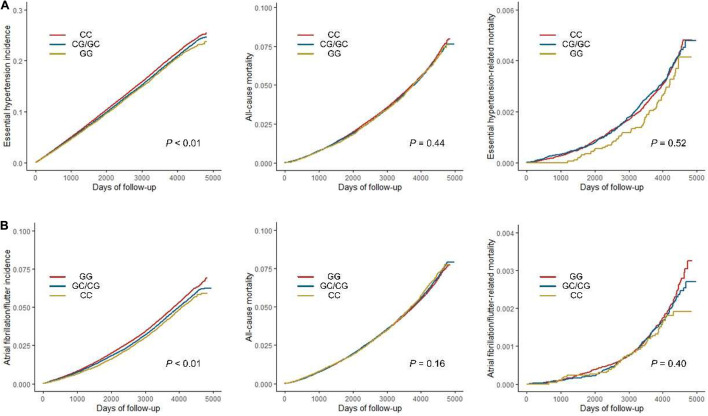
Survival analyses of essential hypertension **(A)** or atrial fibrillation/flutter **(B)** incidence, all-cause and disease-related mortality by genotypes of rs1801253 **(A)** or rs4484922 **(B)**.

**FIGURE 4:**
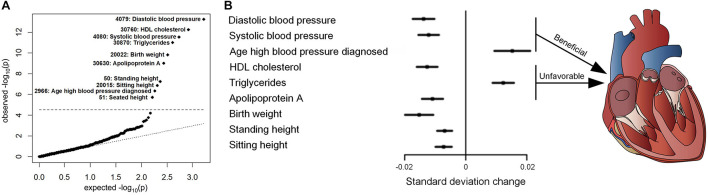
PheWAS of rs1801253 in UKBB. **(A)** QQ plot. The black dashed line represents the Bonferroni-corrected threshold (*p* = 0.05/1,646 = 3.04 × 10^– 5^). The number indicates the field ID of variables in UKBB. **(B)** Forest plot of variables that were significantly associated with rs1801253. The estimates reflect the standard deviation change in the inverse ranked transformed variables per increase of rs1801253 G allele.

To further investigate the effects of variants in replicated associations on CVDs incidence and mortality, survival analyses were performed (Figure 3). The results consistently revealed that rs1801253 and rs4484922 were associated with the incidences of essential hypertension and atrial fibrillation/flutter, respectively, but not all-cause mortality or mortality-related to these diseases (Figure 3). We next studied whether these two variants were correlated to other risk factors that could potentially alter the incidence of their associated clinical outcomes. One thousand six hundred forty-six variables, including 30 biochemistry markers, were screened by PHESANT. Besides the known association between rs1801253 and blood pressure, we also reported a novel association of the blood pressure-lowering G allele of rs1801253 with a delayed onset of hypertension (Figure 4 and Supplementary Table 8). For rs4484922, no association survived Bonferroni or false discovery rate (FDR) correction, but this variant displayed nominal evidence of association with multiple electrocardiogram (ECG) parameters, such as QRS duration (Supplementary Table 7).

## Discussion

The excitability and contraction of cardiomyocytes are largely regulated by Ca^2+^. Ca^2+^ not only participate in the normal myocardial contraction but also enroll in the occurrence of CVDs. Indeed, accumulating evidence suggests a significant impact of genetic polymorphisms and mutations of Ca^2+^ signaling-related genes on CVDs pathogenesis. For example, a literature review evaluated and summarized the effect of 53 SNPs on sudden cardiac death (SCD) and found that rs6684209, rs3814843, and rs35594137 in CASQ2, CALM1, and GJA5, respectively, had the most significant association with SCD (Tamariz et al., 2019). Furthermore, mutations of RYR2, which is responsible for SR Ca^2+^ release, are associated with various CVDs, including dilated cardiomyopathy and hypertrophic cardiomyopathy (Bhuiyan et al., 2007; Tang et al., 2012). Mutation of PLN also induced dilated cardiomyopathy (van der Zwaag et al., 2012). Experiments with human induced pluripotent stem cell (hiPSC) with inserted PLN R9C mutations indicated these cells had a weak response to isoproterenol compared to wild type cells and displayed abnormal Ca^2+^ handling properties, reflecting a pathological state (Ceholski et al., 2018). Moreover, abnormal Ca^2+^ handling in failing cardiomyocytes could promote arrhythmia, and a genetic variant in SERCA2 that is responsible for SR Ca^2+^ uptake has been linked to a decreased arrhythmia risk in patients with heart failure (Francia et al., 2013). GWAS or PheWAS has been widely applied to identify genotype–phenotype associations. However, strict control of false positives by multiple testing corrections in these unbiased estimations may result in the missing of true positive correlation (Johnson et al., 2011). To reduce the multiple testing burden, we included a predefined subset of genotypes (11,274 genetic variants of calcium signaling-related genes) and phenotypes (118 diseases of the circulatory system) based on *a priori* hypotheses that genetic variations of genes related to cardiac Ca^2+^ handling play an essential role in the pathogenesis of a wide range of CVDs. For the identified genotype–clinical outcome association, we employed PHESANT to investigate the potential effect of SNPs on cardiovascular risk factors, including blood pressure and biochemical indexes, which helps to illustrate the mechanisms underlying the development and progression of heart diseases.

Hypertension is a prevalent disease that contributes to a significant portion of premature deaths globally (Surendran et al., 2016). Abnormal increase of blood pressure is a known risk factor for multiple CVDs, such as coronary heart disease and stroke (Surendran et al., 2016), and interventions in blood pressure benefit cardiovascular health (Patel et al., 2016). The heritability of blood pressure can reach 30–50%, highlighting the role of genetic variation in blood pressure determination (Hoffmann et al., 2017). Adrenergic receptors, encoded by ADRB gene, belong to the G-protein-coupled receptors family and play a vital role in regulating myocardial contraction and heart rate (Johnson et al., 2011). The rs1801253 genetic variant in ADRB1 gene leads to arginine to glycine replacement at position 389 of β1 adrenergic receptor (Arg389Gly). The location of Arg389Gly polymorphism is proposed to be a binding domain of Gs-protein (Peng et al., 2009). *In vitro* experiments suggested a higher level of adenylyl cyclase activation in cells with Arg389 β1 adrenergic receptor upon isoprenaline treatment (Rathz et al., 2003). Thus, the enhanced activity caused by Arg389 variant may contribute to a higher cardiac output, which, together with peripheral resistance, determines blood pressure (Peng et al., 2009). Furthermore, polymorphisms in ADRB1 also alter plasma renin activity, which is essential for blood pressure regulation (Petersen et al., 2012). Indeed, the association between Arg389Gly polymorphism and blood pressure/hypertension has been reported across a wide range of ethnicities. For example, the rs1801253 variant was associated with the risk of essential hypertension in a study based on the Chinese Han population (Peng et al., 2009). In a population from Mexico City, participants with homozygous C allele in rs1801253 were more likely to have increased diastolic pressure (Burguete-Garcia et al., 2014). Our results in UKBB with a large sample size consistently revealed that the G allele of rs1801253 was associated with lower diastolic/systolic blood pressure and incidence of hypertension (Figure 4 and Table 1). Moreover, our PheWAS approach identified a delayed onset of hypertension in patients with GG genotype (diagnosed age: 50.99 ± 9.88 years) compared to those with CC genotype (diagnosed age: 50.58 ± 9.94 years) (Figure 4 and Supplementary Table 8). For lipid metabolism traits, the published GWAS meta-analyses from the global lipids genetics consortium (GLGC) indicated that the G allele of rs1801253 was associated with an increased level of triglycerides (TG) and decreased level of high-density lipoprotein (HDL) cholesterol (Willer et al., 2013), which was consistent with our observations (Supplementary Tables 8, 9). We additionally reported that the level of apolipoprotein A, as protein carried in HDL cholesterol, was decreased in participants with G allele (Figure 4 and Supplementary Table 8). Thus, the heart-beneficial blood pressure-lowering rs1801253 G allele is also associated with unfavorable lipid metabolism that is linked to an increased risk of developing CVDs. These double-edged sword effects of Arg389Gly polymorphism may be attributed to its association with fetal growth reflected by birth weight (Horikoshi et al., 2013), which was also replicated in our analyses that G allele of rs1801253 was correlated with a lower birth weight (Figure 4 and Supplementary Table 8). It is worthy of note that when applying the less conservative FDR correction for multiple comparisons, rs1801253 was associated with additional variables, such as apolipoprotein B, pulse rate, and mean sphered cell volume, reflecting the pleiotropy of this variant (Supplementary Table 10).

Arrhythmia is characterized by abnormal heartbeat caused by the dysregulation of electrical impulse generation or conduction (Kennedy et al., 2016). Calcium-related proteins affect cardiac electrophysiology by changing either the electrophysiological properties of cells or heart tissue structure (Kolder et al., 2012). The prolongation of the QT interval measured non-invasively by ECG can potentially promote ventricular arrhythmias, and the effects of genetic polymorphisms in Ca^2+^-handling proteins in myocardial repolarization reflected by the QT interval has been reported (Arking et al., 2014). Arrhythmias is a well-known risk factor for SCD. The correlation between Ca^2+^ and SCD has been proved in an animal model, in which a high level of intracellular Ca^2+^ promoted malignant arrhythmias (Billman et al., 1991). The genetic polymorphisms having the most significant associations with SCD are also from the genes of Ca^2+^-handling proteins responsible for EC coupling (Tamariz et al., 2019). In chronic heart failure patients from the Chinese Han population, both rs3814843 and rs361508, located on CALM1 and triadin (TRDN), respectively, were associated with increased risk of SCD as examined by survival analysis, suggesting common variants in Ca^2+^-handling proteins played an important role in the development of SCD during chronic heart failure (Laver et al., 2015). Cardiac calsequestrin 2, encoded by CASQ2 gene, has a high capacity for Ca^2+^ binding and is responsible for Ca^2+^ storage in SR (Yano and Zarain-Herzberg, 1994). Calsequestrin can also form a complex with RYR2 to modulate SR Ca^2+^ release (Flores et al., 2018). It has been known that mutations in CASQ2 lead to catecholaminergic polymorphic ventricular tachycardia (CPVT), which may trigger cardiac arrest (Faggioni et al., 2012). For the common variants in CASQ2, the C allele of non-synonymous variant rs4074536 was associated with decreased QRS duration in participants of European ancestry (Prins et al., 2018). In the Chinese Han population, a cohort study with 379 participants free of CVDs indicated rs4074536 was associated with the PR interval in ECG. More specifically, PR interval prolongation in individuals with CC genotype compared to those with non-CC genotypes was statistically significant (Li et al., 2020). Using a large sample from UKBB, we consistently found the suggestive association of rs4074536, or its proxy SNPs rs4484922 and rs3810998, with atrial fibrillation/flutter (Table 1 and Supplementary Table 4). Moreover, participants with GG genotype had a prolonged QRS duration compared to those with CC genotype (89.00 ± 14.01 vs. 88.48 ± 14.44 ms) (Supplementary Table 7), which was in accordance with the notion that each copy of C allele in rs4074536 was associated with less than 1 ms shortening of QRS duration (Sotoodehnia et al., 2010). Interestingly, our PheWAS approach revealed that rs4484922 showed nominal evidence of association with multiple parameters of carotid intima-medial thickness (cIMT) ultrasound measurement (Supplementary Table 7), and the elevated cIMT observed in individuals with CC genotype may increase the risk of atherosclerosis, which is an underlying cause of multiple CVDs (Strawbridge et al., 2020).

We found suggestive association of rs79613429 located in the intron of RYR2 gene with precordial pain (*p* = 4.39 × 10^–7^). Genetic variants located in introns are well recognized to regulate mRNA splicing and gene expression (Wang and Cooper, 2007; Faraday et al., 2011). However, how these variants lead to an altered risk of clinical outcomes needs to be further elucidated. The major strength of our study is that multiple genotype–clinical outcome associations are systematically tested to investigate the role of calcium signaling-related genetic variants in CVDs. Furthermore, we performed the analyses in a large sample from the UKBB to avoid the decreased power in detecting association between CVDs and common variants with MAF below 5% (Altshuler et al., 2008). For the classification of clinical outcomes in electronic health records, we employed the phecode system that is more representative of independent phenotypes compared to ICD codes (Denny et al., 2010; Rahimi et al., 2019; King et al., 2020). Moreover, excluding participants with phecodes/diseases related to the investigating phecode/disease in the analysis prevented the contamination of the control population by cases, leading to an increased statistical power (Wei et al., 2017). Our genetic variant-based PheWAS is also advantageous because the genotype is fixed at birth and the results are less susceptible to reverse causality (Shen et al., 2020). The present study also has several limitations. First, using a single SNP in the correlation analyses can predict only a small effect size of the association (Cai et al., 2018). Second, the novel associations observed in our study are needed to be replicated in independent datasets of similar ancestry to enhance their credibility. Moreover, these associations were solely based on the European population, and replication in non-European populations will further validate our results. Also, cell and animal-based experiments could be applied to examine the functions of the reported variants to discover novel biological mechanisms.

## Conclusion

Multiple associations of genetic variants in calcium signaling-related genes with CVDs and cardiovascular risk factors have been identified, highlighting genetic testing as a useful method for disease diagnosis and prevention.

## Data Availability Statement

The data used in this study is publicly available and can be obtained from United Kingdom Biobank (http://www.ukbiobank.ac.uk).

## Ethics Statement

All participants provided written informed consent, and the United Kingdom Biobank is ethically approved by the National Research Ethics Service (ref 11/NW/0382).

## Author Contributions

SL designed the study. ZJ and ZZ performed the statistical analyses. SL, ZZ, and YL drafted the manuscript. MY and TY critically reviewed the manuscript. All authors read and approved the final manuscript.

## Conflict of Interest

The authors declare that the research was conducted in the absence of any commercial or financial relationships that could be construed as a potential conflict of interest.

## Publisher’s Note

All claims expressed in this article are solely those of the authors and do not necessarily represent those of their affiliated organizations, or those of the publisher, the editors and the reviewers. Any product that may be evaluated in this article, or claim that may be made by its manufacturer, is not guaranteed or endorsed by the publisher.

## Abbreviations

EC, coupling: excitation–contraction coupling
RYRs: ryanodine receptors
SR: sarcoplasmic reticulum
SERCA: sarco/endoplasmic reticulum Ca^2+^-ATPase
NCX: sodium–calcium exchanger
CALM: calmodulin
PLN: phospholamban
CVDs: cardiovascular diseases
DAD: delayed after-depolarization
EAD: early after-depolarization
CPVT: catecholaminergic polymorphic ventricular tachycardia
UKBB: United Kingdom Biobank
PheWAS: phenome-wide association study
HWE: Hardy–Weinberg equilibrium
MAF: minor allele frequency
ICD: the International Classification of Diseases
PCs: principal components
PHESANT: phenome scan analysis tool
LD: linkage disequilibrium
FDR: false discovery rate
ECG: electrocardiogram
SCD: sudden cardiac death
hiPSC: human induced pluripotent stem cell
GWAS: genome-wide association studies
TRDN: triadin
GLGC: global lipids genetics consortium
cIMT: carotid intima-medial thickness
BMI: body mass index
HDL: high-density lipoprotein
TG: triglycerides
OR: odds ratio
N: number
SD: standard deviation
95% CI: 95% confidence interval.
