# Supplementary material for: Association Study of Genetic Variants in Calcium Signaling-Related Genes With Cardiovascular Diseases
Source: Front Cell Dev Biol. 2021 Nov 29;9:642141. doi: 10.3389/fcell.2021.642141 (PMC8666440; doi:10.3389/fcell.2021.642141)
Supplement: Supplementary file 1 [file Table_1.docx]

Supplementary Table 1. The number of analyzed genetic variants for each of the nine calcium signaling-related genes.

| Gene | Chr | No. of SNPs | No. of independent SNPs at gene level | *P*-value thresholds for suggestive association |  |
| --- | --- | --- | --- | --- | --- |
| CASQ2 | 1 | 422 | 69 | 7.55E-06 |  |
| RYR2 | 1 | 3863 | 915 | 5.69E-07 |  |
| SLC8A1 | 2 | 2120 | 556 | 9.37E-07 |  |
| PLN | 6 | 29 | 13 | 4.01E-05 |  |
| TRDN | 6 | 1743 | 355 | 1.47E-06 |  |
| ADRB1 | 10 | 6 | 4 | 1.30E-04 |  |
| CACNA1C | 12 | 2849 | 822 | 6.34E-07 |  |
| ATP2A2 | 12 | 214 | 44 | 1.18E-05 |  |
| CALM1 | 14 | 28 | 12 | 4.34E-05 |  |
| Abbreviation: Chr: chromosome; SNP: single nucleotide polymorphism; CASQ2: calsequestrin 2; RYR2: ryanodine receptor 2; SLC8A1: sodium/calcium exchanger 1; PLN: phospholamban; TRDN: triadin; ADRB1: beta-1 adrenergic receptor; CACNA1C: calcium voltage-gated channel subunit alpha1 C; ATP2A2: sarcoplasmic/endoplasmic reticulum calcium ATPase 2; CALM1: calmodulin-1. | | | | | |

Supplementary Table 2. Descriptive characteristics of the study population (N=308,366).

| Characteristics | Mean/N (SD/%) |
| --- | --- |
| Age, years (SD) | 56.6 (8.0) |
| Sex, female (%) | 165,739 (53.8%) |
| TDI (SD) | -1.5 (3.0) |
| Education, have a degree (%) | 107,152 (34.8%) |
| BMI, ≥25 (%) | 203,814 (66.1%) |
| Smoking, yes (%) | 141,312 (45.8%) |
| Drinking, yes (%) | 221,705 (71.9%) |
| Abbreviation: N: number; SD: standard deviation; TDI, Townsend Deprivation Index; BMI, Body Mass Index. | |

Supplementary Table 3. Description and number of participants analyzed for each of the 118 CVDs/phecodes.

| Phecode | Description | Total (N) | | |  | Cases (N) | | |
| --- | --- | --- | --- | --- | --- | --- | --- | --- |
|  |  | Minimum | Median | Maximum |  | Minimum | Median | Maximum |
| 394 | Rheumatic disease of the heart valves | 275446 | 304553 | 305973 |  | 7191 | 7972 | 8008 |
| 394.2 | Mitral valve disease | 272432 | 301248 | 302655 |  | 4203 | 4669 | 4690 |
| 394.3 | Aortic valve disease | 269955 | 298533.5 | 299928 |  | 1750 | 1954 | 1963 |
| 394.7 | Disease of tricuspid valve | 270273 | 298866 | 300261 |  | 2036 | 2286 | 2296 |
| 395 | Heart valve disorders | 274005 | 302974.5 | 304389 |  | 5766 | 6395 | 6424 |
| 395.1 | Nonrheumatic mitral valve disorders | 272345 | 301146 | 302552 |  | 4109 | 4566 | 4587 |
| 395.2 | Nonrheumatic aortic valve disorders | 268574 | 297019.5 | 298406 |  | 385 | 439 | 441 |
| 395.3 | Nonrheumatic tricuspid valve disorders | 268389 | 296813 | 298197 |  | 202 | 231 | 232 |
| 395.4 | Nonrheumatic pulmonary valve disorders | 268434 | 296867.5 | 298253 |  | 250 | 287 | 288 |
| 395.6 | Heart valve replaced | 269952 | 298540 | 299933 |  | 1753 | 1959 | 1968 |
| 396 | Abnormal heart sounds | 269888 | 298490 | 299883 |  | 1702 | 1909 | 1918 |
| 401 | Hypertension | 277603 | 306933 | 308366 |  | 73988 | 81842 | 82221 |
| 401.1 | Essential hypertension | 277496 | 306816.5 | 308249 |  | 73875 | 81726 | 82104 |
| 401.2 | Hypertensive heart and/or renal disease | 204679 | 226416 | 227475 |  | 1173 | 1324 | 1330 |
| 401.22 | Hypertensive chronic kidney disease | 204504 | 226217 | 227275 |  | 993 | 1125 | 1130 |
| 411 | Ischemic Heart Disease | 276594 | 305852 | 307279 |  | 28391 | 31445 | 31589 |
| 411.1 | Unstable angina (intermediate coronary syndrome) | 252091 | 278752.5 | 280052 |  | 3884 | 4342 | 4362 |
| 411.2 | Myocardial infarction | 259817 | 287319.5 | 288659 |  | 11650 | 12910 | 12969 |
| 411.3 | Angina pectoris | 262282 | 290018 | 291371 |  | 14064 | 15609 | 15681 |
| 411.4 | Coronary atherosclerosis | 266996 | 295255 | 296632 |  | 18822 | 20847 | 20942 |
| 411.41 | Aneurysm and dissection of heart | 248992 | 275329 | 276613 |  | 821 | 919 | 923 |
| 411.8 | Other chronic ischemic heart disease, unspecified | 262696 | 290496 | 291849 |  | 14494 | 16085 | 16159 |
| 411.9 | Other acute and subacute forms of ischemic heart disease | 249795 | 276236 | 277525 |  | 1623 | 1827 | 1835 |
| 414 | Other forms of chronic heart disease | 251592 | 278206 | 279504 |  | 3402 | 3797 | 3814 |
| 415 | Pulmonary heart disease | 274154 | 303114 | 304530 |  | 5026 | 5592 | 5618 |
| 415.2 | Chronic pulmonary heart disease | 270224 | 298811 | 300207 |  | 1152 | 1289 | 1295 |
| 415.21 | Primary pulmonary hypertension | 269431 | 297945.5 | 299339 |  | 374 | 425 | 427 |
| 416 | Cardiomegaly | 273075 | 301965 | 303376 |  | 4010 | 4444 | 4464 |
| 418 | Nonspecific chest pain | 275254 | 304341 | 305764 |  | 24283 | 26880 | 27006 |
| 418.1 | Precordial pain | 254910 | 281823.5 | 283138 |  | 3915 | 4360 | 4380 |
| 420 | Carditis | 276236 | 305420.5 | 306847 |  | 2461 | 2738 | 2750 |
| 420.2 | Pericarditis | 275305 | 304408 | 305829 |  | 1543 | 1724 | 1732 |
| 420.21 | Acute pericarditis | 273995 | 302915 | 304320 |  | 200 | 222 | 223 |
| 420.3 | Endocarditis | 274628 | 303645 | 305062 |  | 858 | 961 | 965 |
| 425 | Cardiomyopathy | 275255 | 304346 | 305767 |  | 1473 | 1663 | 1670 |
| 425.1 | Primary/intrinsic cardiomyopathies | 275214 | 304303.5 | 305724 |  | 1435 | 1620 | 1627 |
| 425.12 | Other hypertrophic cardiomyopathy | 273971 | 302927.5 | 304342 |  | 212 | 244 | 245 |
| 426 | Cardiac conduction disorders | 257773 | 285072 | 286405 |  | 9449 | 10494 | 10542 |
| 426.2 | Atrioventricular [AV] block | 251522 | 278182.5 | 279482 |  | 3237 | 3603 | 3619 |
| 426.21 | First degree AV block | 249983 | 276507 | 277799 |  | 1725 | 1927 | 1936 |
| 426.23 | Second degree AV block | 248878 | 275291.5 | 276578 |  | 631 | 712 | 715 |
| 426.24 | Atrioventricular block, complete | 249112 | 275544 | 276833 |  | 855 | 966 | 970 |
| 426.3 | Bundle branch block | 253262 | 280126 | 281435 |  | 4979 | 5547 | 5572 |
| 426.31 | Right bundle branch block | 250716 | 277307.5 | 278604 |  | 2454 | 2729 | 2741 |
| 426.32 | Left bundle branch block | 250751 | 277374.5 | 278672 |  | 2507 | 2796 | 2809 |
| 426.4 | Anomalous atrioventricular excitation | 267027 | 275803 | 276063 |  | 200 | 200 | 200 |
| 426.9 | Cardiac pacemaker/device in situ | 251537 | 278249.5 | 279549 |  | 3290 | 3669 | 3686 |
| 426.91 | Cardiac pacemaker in situ | 251320 | 278010.5 | 279310 |  | 3074 | 3431 | 3447 |
| 427 | Cardiac dysrhythmias | 272949 | 301837 | 303244 |  | 24614 | 27255 | 27381 |
| 427.1 | Paroxysmal tachycardia, unspecified | 251673 | 278371.5 | 279673 |  | 3409 | 3793 | 3810 |
| 427.11 | Paroxysmal supraventricular tachycardia | 250726 | 277319 | 278615 |  | 2458 | 2740 | 2752 |
| 427.12 | Paroxysmal ventricular tachycardia | 249280 | 275745.5 | 277035 |  | 1037 | 1167 | 1172 |
| 427.2 | Atrial fibrillation and flutter | 261779 | 289473.5 | 290823 |  | 13439 | 14892 | 14960 |
| 427.21 | Atrial fibrillation | 251262 | 277921 | 279219 |  | 2990 | 3341 | 3356 |
| 427.22 | Atrial flutter | 248937 | 275352 | 276638 |  | 680 | 772 | 775 |
| 427.3 | Other specified cardiac dysrhythmias | 252401 | 279172.5 | 280477 |  | 4130 | 4593 | 4614 |
| 427.4 | Cardiac arrest and ventricular fibrillation | 249707 | 276205 | 277497 |  | 1443 | 1627 | 1634 |
| 427.41 | Ventricular fibrillation and flutter | 248622 | 275016.5 | 276302 |  | 372 | 437 | 439 |
| 427.42 | Cardiac arrest | 249511 | 275983.5 | 277274 |  | 1252 | 1405 | 1411 |
| 427.5 | Arrhythmia (cardiac) NOS | 249335 | 275806 | 277096 |  | 1095 | 1228 | 1233 |
| 427.6 | Premature beats | 249391 | 275877.5 | 277167 |  | 1161 | 1298 | 1304 |
| 427.7 | Tachycardia NOS | 251077 | 277738 | 279037 |  | 2845 | 3159 | 3174 |
| 427.8 | Sinoatrial node dysfunction (Bradycardia) | 248845 | 275256 | 276542 |  | 590 | 676 | 679 |
| 427.9 | Palpitations | 252066 | 278822.5 | 280126 |  | 3803 | 4243 | 4263 |
| 428 | Congestive heart failure; nonhypertensive | 276850 | 306103.5 | 307531 |  | 7312 | 8109 | 8145 |
| 428.1 | Congestive heart failure (CHF) NOS | 272623 | 301482 | 302888 |  | 3130 | 3486 | 3502 |
| 428.2 | Heart failure NOS | 275262 | 304367 | 305786 |  | 5727 | 6372 | 6400 |
| 429 | Ill-defined descriptions and complications of heart disease | 270300 | 298946.5 | 300343 |  | 842 | 953 | 957 |
| 429.2 | Abnormal function study of cardiovascular system | 270074 | 298695 | 300090 |  | 615 | 701 | 704 |
| 429.3 | Symptoms involving cardiovascular system | 269657 | 298241 | 299633 |  | 207 | 246 | 247 |
| 430 | Intracranial hemorrhage | 268182 | 296616 | 298001 |  | 1950 | 2184 | 2194 |
| 430.1 | Subarachnoid hemorrhage | 266961 | 295260.5 | 296638 |  | 735 | 828 | 831 |
| 430.2 | Intracerebral hemorrhage | 267100 | 295428.5 | 296807 |  | 881 | 995 | 1000 |
| 430.3 | Subdural hemorrhage | 266560 | 294835 | 296211 |  | 353 | 402 | 404 |
| 433 | Cerebrovascular disease | 276388 | 305594 | 307021 |  | 10070 | 11163 | 11214 |
| 433.1 | Occlusion and stenosis of precerebral arteries | 267777 | 296175 | 297557 |  | 1552 | 1742 | 1750 |
| 433.12 | Cerebral atherosclerosis | 266516 | 294782 | 296158 |  | 300 | 349 | 351 |
| 433.2 | Occlusion of cerebral arteries | 270906 | 299580 | 300980 |  | 4628 | 5149.5 | 5173 |
| 433.21 | Cerebral artery occlusion, with cerebral infarction | 268399 | 296859 | 298245 |  | 2180 | 2427 | 2438 |
| 433.3 | Cerebral ischemia | 270069 | 298678 | 300072 |  | 3822 | 4246 | 4265 |
| 433.31 | Transient cerebral ischemia | 268313 | 296768.5 | 298153 |  | 2097 | 2335 | 2346 |
| 433.5 | Cerebral aneurysm | 266576 | 294846 | 296221 |  | 364 | 412 | 414 |
| 433.8 | Late effects of cerebrovascular disease | 267697 | 296073.5 | 297457 |  | 1468 | 1642 | 1650 |
| 440 | Atherosclerosis | 269531 | 298022 | 299413 |  | 1233 | 1379 | 1385 |
| 440.2 | Atherosclerosis of the extremities | 269186 | 297639 | 299028 |  | 892 | 995 | 1000 |
| 441 | Vascular insufficiency of intestine | 269039 | 297480.5 | 298870 |  | 745 | 838 | 842 |
| 441.1 | Acute vascular insufficiency of intestine | 268621 | 297017.5 | 298405 |  | 326 | 375 | 377 |
| 442 | Other aneurysm | 270658 | 299262 | 300658 |  | 2344 | 2618 | 2630 |
| 442.1 | Aortic aneurysm | 270172 | 298730 | 300124 |  | 1870 | 2087 | 2096 |
| 442.11 | Abdominal aortic aneurysm | 269489 | 297967.5 | 299358 |  | 1186 | 1324 | 1330 |
| 442.3 | Aneurysm of artery of lower extremity | 268829 | 296992.5 | 298237 |  | 200 | 208 | 209 |
| 442.8 | Aneurysm of other specified artery | 268542 | 296931.5 | 298318 |  | 244 | 289 | 290 |
| 443 | Peripheral vascular disease | 272719 | 301532.5 | 302940 |  | 4409 | 4890 | 4912 |
| 443.1 | Raynaud's syndrome | 269686 | 298182.5 | 299574 |  | 1366 | 1539 | 1546 |
| 443.7 | Peripheral angiopathy in diseases classified elsewhere | 268730 | 297139 | 298525 |  | 437 | 495 | 497 |
| 443.9 | Peripheral vascular disease, unspecified | 271129 | 299789.5 | 301189 |  | 2832 | 3146 | 3161 |
| 444 | Arterial embolism and thrombosis | 269187 | 297658.5 | 299048 |  | 902 | 1015 | 1020 |
| 444.1 | Arterial embolism and thrombosis of lower extremity artery | 268833 | 297259 | 298647 |  | 544 | 616 | 619 |
| 446 | Polyarteritis nodosa and allied conditions | 269228 | 297685.5 | 299075 |  | 931 | 1043 | 1047 |
| 446.5 | Giant cell arteritis | 268779 | 297196 | 298583 |  | 493 | 553 | 555 |
| 446.9 | Arteritis NOS | 268586 | 296976 | 298362 |  | 292 | 333 | 334 |
| 447 | Other disorders of arteries and arterioles | 269639 | 298153 | 299546 |  | 1349 | 1511 | 1518 |
| 447.1 | Stricture of artery | 269142 | 297603 | 298994 |  | 852 | 962 | 966 |
| 450 | Noninfectious disorders of lymphatic channels | 277603 | 306933 | 308366 |  | 1052 | 1181 | 1186 |
| 451 | Phlebitis and thrombophlebitis | 244763 | 270635.5 | 271901 |  | 3774 | 4217 | 4237 |
| 451.2 | Phlebitis and thrombophlebitis of lower extremities | 244448 | 270283.5 | 271547 |  | 3457 | 3865 | 3883 |
| 452 | Other venous embolism and thrombosis | 241631 | 267196.5 | 268446 |  | 684 | 779 | 782 |
| 454 | Varicose veins | 250682 | 277176.5 | 278472 |  | 9682 | 10759 | 10808 |
| 454.1 | Varicose veins of lower extremity | 250236 | 276676.5 | 277970 |  | 9222 | 10259 | 10306 |
| 454.11 | Varicose veins of lower extremity, symptomtic | 241791 | 267335 | 268586 |  | 809 | 918 | 922 |
| 455 | Hemorrhoids | 264629 | 292587 | 293954 |  | 23635 | 26168.5 | 26290 |
| 456 | Chronic venous insufficiency [CVI] | 241284 | 266791 | 268039 |  | 328 | 373 | 375 |
| 458 | Hypotension | 261030 | 288665 | 290013 |  | 8825 | 9805 | 9851 |
| 458.1 | Orthostatic hypotension | 254504 | 281458.5 | 282773 |  | 2333 | 2599 | 2611 |
| 458.2 | Iatrogenic hypotension | 252431 | 279195 | 280499 |  | 294 | 336 | 337 |
| 458.9 | Hypotension NOS | 257678 | 284990 | 286323 |  | 5493 | 6132 | 6161 |
| 459 | Other disorders of circulatory system | 271152 | 299830.5 | 301231 |  | 18935 | 20971 | 21069 |
| 459.9 | Circulatory disease NEC | 270988 | 299646 | 301046 |  | 18766 | 20787 | 20884 |

Supplementary Table 4. LD (in term of R^2^) of identified SNPs.

| **CASQ2** | | |
| --- | --- | --- |
| **SNP Pair** | | **R^2^** |
| rs4484922 | rs4074536 | 1.00 |
| rs4484922 | rs3810998 | 0.99 |
| rs4074536 | rs3810998 | 0.99 |
| **RYR2** | | |
| **SNP Pair** | | **R^2^** |
| rs116662749 | rs142214989 | 0.95 |
| rs116662749 | rs80129764 | 0.95 |
| rs116662749 | rs79613429 | 0.94 |
| rs142214989 | rs80129764 | 1.00 |
| rs142214989 | rs79613429 | 0.99 |
| rs80129764 | rs79613429 | 0.99 |
| **TRDN** | | |
| **SNP Pair** | | **R^2^** |
| rs62420492 | rs62420493 | 1.00 |
| Abbreviation: SNP: single nucleotide polymorphism; CASQ2: calsequestrin 2; RYR2: ryanodine receptor 2; TRDN: triadin. | | |

Supplementary Table 5. The associations between SNPs and CVDs after multivariable adjustment.

| Gene | SNP | Phenotype | OR (95% CI) | *P* |
| --- | --- | --- | --- | --- |
| ADRB1 | rs1801253 | Hypertension | 0.95 (0.93-0.96) | 4.77E-15 |
| ADRB1 | rs1801253 | Essential hypertension | 0.95 (0.93-0.96) | 4.84E-15 |
| CASQ2 | rs4484922 | Atrial fibrillation and flutter | 0.94 (0.91-0.96) | 2.86E-06 |
| RYR2 | rs79613429 | Precordial pain | 1.22 (1.13-1.32) | 5.22E-07 |
| TRDN | rs62420492 | Essential hypertension | 0.96 (0.94-0.98) | 1.39E-05 |
| TRDN | rs62420492 | Hypertension | 0.96 (0.94-0.98) | 1.43E-05 |
| Age, sex and the status of overweight (BMI≥25 kg/m^2^), smoking or drinking were treated as covariables,  and the first five principal components (PCs) were also adjusted for population structure.  Abbreviation: SNP: single nucleotide polymorphism; OR: odds ratio; 95% CI: 95% confidence interval;  ADRB1: beta-1 adrenergic receptor; CASQ2: calsequestrin 2; RYR2: ryanodine receptor 2; TRDN: triadin. | | | | |

Supplementary Table 6. The reported associations of identified SNPs (and their proxies) with phenotypes in the GWAS catalog.

| DISEASE/TRAIT | | | | | | SNP-RISK ALLELE | | OR or BETA | 95% CI | PUBMEDID |
| --- | --- | --- | --- | --- | --- | --- | --- | --- | --- | --- |
| rs1801253 | | | | | | | | | | |
| Birth weight | | | | | | rs1801253-G | | 0.041 | [0.027-0.055] gram decrease | 23202124 |
| Systolic blood pressure | | | | | | rs1801253-C | | 0.0235 | unit increase | 27618447 |
| Diastolic blood pressure | | | | | | rs1801253-G | | 0.183 | unit decrease | 27841878 |
| Systolic blood pressure | | | | | | rs1801253-G | | 0.273 | unit decrease | 27841878 |
| Offspring birth weight | | | | | | rs1801253-C | | 0.021484 | [0.015-0.028] unit increase | 31043758 |
| Systolic blood pressure | | | | | | rs1801253-G | | 0.2724 | [0.2-0.35] mmHg increase | 30578418 |
| Birth weight | | | | | | rs1801253-C | | 0.028 | [0.02-0.036] z score increase | 31097437 |
| Cardiovascular disease | | | | | | rs1801253-? | |  |  | 30595370 |
| Height | | | | | | rs1801253-? | |  |  | 30595370 |
| Medication use (agents acting on the renin-angiotensin system) | | | | | | rs1801253-G | | 0.050945096 | [0.037-0.065] unit decrease | 31015401 |
| rs1801253's proxy SNP rs2773469 (R^2^=0.834) rs1801253(C) and (G) allele is correlated with rs2773469(G) and (A) allele, respectively | | | | | | | | | | |
| Triglyceride levels | | | | | | rs2773469-A | | 0.0186292 | [0.014-0.023] unit increase | 32203549 |
| rs1801253's proxy SNP rs740746 (R^2^=0.82)  rs1801253(C) and (G) allele is correlated with rs740746(A) and (G) allele, respectively | | | | | | | | | | |
| Diastolic blood pressure | | | | | | rs740746-A | | 0.32 | [0.24-0.4] mm Hg increase | 27618452 |
| Systolic blood pressure | | | | | | rs740746-A | | 0.486 | [0.35-0.62] mm Hg increase | 27618452 |
| Birth weight (MTAG) | | | | | | rs740746-A | | 0.037240412 | [0.029-0.045] unit increase | 31681408 |
| Birth length (MTAG) | | | | | | rs740746-A | | 0.051283136 | [0.039-0.063] unit increase | 31681408 |
| Infant head circumference (MTAG) | | | | | | rs740746-A | | 0.07175104 | [0.047-0.096] z score increase | 31681408 |
| Lipid traits (pleiotropy) (HIPO component 1) | | | | | | rs740746-? | |  |  | 30289880 |
| rs1801253's proxy SNP rs7076938 (R^2^=0.806) rs1801253(C) and (G) allele is correlated with rs7076938(T) and (C) allele, respectively | | | | | | | | | | |
| Mean arterial pressure | | | | | | rs7076938-C | | 0.30268204 | [0.2-0.4] unit decrease | 27618448 |
| Birth weight | | | | | | rs7076938-T | | 0.0349 | [0.027-0.043] unit increase | 27680694 |
| High density lipoprotein cholesterol levels | | | | | | rs7076938-T | | 0.017042626 | [0.013-0.021] unit increase | 29083408 |
| Birth weight | | | | | | rs7076938-T | | 0.032051 | [0.026-0.038] unit increase | 31043758 |
| Medication use (diuretics) | | | | | | rs7076938-C | | 0.053165883 | [0.036-0.071] unit decrease | 31015401 |
| rs4484922 | | | | | | | | | | |
| Atrial fibrillation | | | | | | rs4484922-G | | 1.07 | [1.05-1.08] | 29892015 |
| Atrial fibrillation | | | | | | rs4484922-G | | 1.07 | [1.05-1.09] | 29892015 |
| rs4074536 | | | | | | | | | | |
| QRS duration | | | | | | rs4074536-C | | 0.309967 | [0.24-0.38] ms decrease | 30012220 |
| QRS duration | | | | | | rs4074536-C | | 0.33213338 | [0.23-0.43] ms decrease | 30012220 |
| QRS duration | | | | | | rs4074536-? | |  |  | 27659466 |
| Results were based on LDtrait Tool (https://ldlink.nci.nih.gov/?tab=ldtrait) search, and R^2^ of proxy SNPs were calculated by LDpair Tool (https://ldlink.nci.nih.gov/?tab=ldpair). | | | | | | | | | | |
| Supplementary Table 7. Mean and SD of QRS duration and cIMT that are associated with rs4484922 in PHESANT.   \|  \|  \|  \| rs4484922 \|  \| \| --- \| --- \| --- \| --- \| --- \| \|  \|  \| GG \| GC/CG \| CC \| \| QRS duration (ms) \| N \| 13,547 \| 11,324 \| 2,359 \| \|  \| Mean (SD) \| 89.00 (14.01) \| 88.84 (14.28) \| 88.48 (14.44) \| \| cIMT mean (μm) \| N \| 14,449 \| 12,143 \| 2,512 \| \|  \| Mean (SD) \| 686.16 (125.14) \| 687.61 (125.16) \| 694.68 (133.33) \| \| Abbreviation: PHESANT: phenome scan analysis tool; SD: standard deviation; cIMT: carotid intima-medial thickness. \| \| \| \| \|   Supplementary Table 8. Mean and SD of the traits that are associated with rs1801253 in PHESANT.   \|  \| \| \| \|  \| \| --- \| --- \| --- \| --- \| --- \| \|  \|  \|  \| rs1801253 \|  \| \|  \|  \| CC \| CG/GC \| GG \| \| Diastolic blood pressure (mmHg) \| N \| 164,721 \| 117,278 \| 20,833 \| \|  \| Mean (SD) \| 82.31 (10.7) \| 82.07 (10.68) \| 81.72 (10.67) \| \| Systolic blood pressure (mmHg) \| N \| 164,718 \| 117,276 \| 20,832 \| \|  \| Mean (SD) \| 139.95 (19.61) \| 139.61 (19.73) \| 139.02 (19.67) \| \| Age high blood pressure diagnosed (years) \| N \| 40,238 \| 27,145 \| 4,609 \| \|  \| Mean (SD) \| 50.58 (9.94) \| 50.98 (9.88) \| 50.99 (9.88) \| \| HDL cholesterol (mmol/L) \| N \| 144,226 \| 102,312 \| 18,192 \| \|  \| Mean (SD) \| 1.46 (0.39) \| 1.45 (0.38) \| 1.45 (0.38) \| \| Triglycerides (mmol/L) \| N \| 157,274 \| 111,833 \| 19,866 \| \|  \| Mean (SD) \| 1.73 (1.01) \| 1.75 (1.03) \| 1.77 (1.03) \| \| Apolipoprotein A (g/L) \| N \| 143,392 \| 101,738 \| 18,095 \| \|  \| Mean (SD) \| 1.55 (0.27) \| 1.54 (0.27) \| 1.54 (0.27) \| \| Birth weight (Kg) \| N \| 94,043 \| 66,910 \| 11,722 \| \|  \| Mean (SD) \| 3.33 (0.66) \| 3.32 (0.66) \| 3.30 (0.66) \| \| Standing height (cm) \| N \| 164,852 \| 117,384 \| 20,841 \| \|  \| Mean (SD) \| 169.01 (9.24) \| 168.81 (9.24) \| 168.83 (9.27) \| \| Sitting height (cm) \| N \| 164,836 \| 117,365 \| 20,839 \| \|  \| Mean (SD) \| 89.47 (4.81) \| 89.38 (4.81) \| 89.36 (4.81) \| \| Abbreviation: PHESANT: phenome scan analysis tool; SD: standard deviation. \| \| \| \| \|   Supplementary Table 9. Associations between rs1801253 and lipid metabolic traits from published genome-wide association study meta-analyses from Global Lipids Genetics Consortium (GLGC). | | | | | | | | | | |
|  | A1 | A2 | beta | se | N | | *P*-value |  |  |  |
| HDL | C | G | 0.0187 | 0.0044 | 157555.07 | | 3.48E-06 |  |  |  |
| LDL | C | G | 0.0060 | 0.0047 | 144391.96 | | 0.30 |  |  |  |
| TC | C | G | 0.0107 | 0.0045 | 157585.03 | | 0.01 |  |  |  |
| TG | G | C | 0.0099 | 0.0042 | 148491.06 | | 0.03 |  |  |  |
| Abbreviation: A1: effect allele; A2: other allele; Beta: effect size; SE: standard error for Beta; N: the number of individuals analyzed. | | | | | | | |  |  |  |

Supplementary Table 10. Associations between rs1801253 and variables reaching 5% FDR significance.

| Variables | N | Beta | Lower | Upper | *P* | *P*_BH-adjusted_ |
| --- | --- | --- | --- | --- | --- | --- |
| Diastolic blood pressure, automated reading | 285,987 | -0.01381 | -0.01741 | -0.01020 | 6.03E-14 | 9.92E-11 |
| HDL cholesterol | 264,730 | -0.01265 | -0.01609 | -0.00921 | 5.79E-13 | 4.77E-10 |
| Systolic blood pressure, automated reading | 285,985 | -0.01215 | -0.01556 | -0.00873 | 2.99E-12 | 1.64E-09 |
| Triglycerides | 288,973 | 0.01228 | 0.00875 | 0.01582 | 9.72E-12 | 4.00E-09 |
| Birth weight | 172,675 | -0.01526 | -0.01993 | -0.01059 | 1.49E-10 | 4.91E-08 |
| Apolipoprotein A | 263,225 | -0.01090 | -0.01440 | -0.00740 | 1.04E-09 | 2.87E-07 |
| Standing height | 303,077 | -0.00691 | -0.00941 | -0.00441 | 5.84E-08 | 1.37E-05 |
| Sitting height | 303,040 | -0.00724 | -0.00994 | -0.00455 | 1.35E-07 | 2.78E-05 |
| Age high blood pressure diagnosed | 71,992 | 0.01525 | 0.00933 | 0.02116 | 4.42E-07 | 8.09E-05 |
| Seated height | 303,040 | -0.00678 | -0.00957 | -0.00399 | 1.88E-06 | 0.00031 |
| Mean sphered cell volume | 289,310 | -0.00739 | -0.01101 | -0.00376 | 6.50E-05 | 0.00972 |
| Forced expiratory volume in 1-second (FEV1), predicted | 98,456 | -0.00510 | -0.00775 | -0.00245 | 0.00016 | 0.02220 |
| Apolipoprotein B | 287,783 | 0.00675 | 0.00310 | 0.01040 | 0.00029 | 0.03669 |
| Pulse rate, automated reading | 285,987 | -0.00661 | -0.01026 | -0.00297 | 0.00038 | 0.04288 |
| Mother's age at death | 176,647 | 0.00803 | 0.00359 | 0.01247 | 0.00039 | 0.04288 |
| FDR: false discovery rate; N: number; Lower: lower bound of 95% confidence interval; Upper: upper bound of 95% confidence interval; BH: Benjamini-Hochberg. | | | | | | |
